# Stress Echocardiography in Aortic Stenosis: From Diagnostic Challenges to Guideline-Endorsed Clinical Applications

**DOI:** 10.3390/jcm14207424

**Published:** 2025-10-21

**Authors:** Roxana Hodas, Călin Pop, Antoniu Octavian Petris

**Affiliations:** 1 Emergency County Hospital Baia Mare, 430130 Baia Mare, Romania; roxana.hodas@gmail.com; 2 2nd Department, Faculty of Nursing and Health Sciences, “Iuliu Hatieganu” University of Medicine, 400349 Cluj-Napoca, Romania; 3 Cardiology Clinic, “Grigore T. Popa” University of Medicine and Pharmacy, 700115 Iaşi, Romania; antoniu.petris@yahoo.ro; 4 “Sf. Spiridon” Clinical County Emergency Hospital, 700111 Iaşi, Romania

**Keywords:** aortic stenosis, stress echocardiography, exercise and dobutamine stress echocardiography, risk stratification, aortic valve replacement

## Abstract

Aortic stenosis (AS) is the most common valvular heart disease in industrialized countries. Stress echocardiography (SE), using either exercise or dobutamine protocols, has emerged as a critical tool to overcome limitations of resting echocardiography, refine risk stratification, and guide the timing of aortic valve replacement. This review synthesizes contemporary evidence on the diagnostic, prognostic, and therapeutic role of SE in AS. Studies from all main databases (2000–2025) were systematically analyzed including prospective studies, consensus statements, and international guidelines. We highlight the physiological rationale, key prognostic markers, applications in asymptomatic severe and low-flow, low-gradient AS, and integration with multimodality imaging. SE is now guideline-endorsed for risk stratification in asymptomatic severe AS and the diagnosis of true severe versus pseudo-severe AS in low-flow, low-gradient disease. Future directions include advanced strain imaging, artificial intelligence, and broader adoption in the transcatheter era.

## 1. Introduction

Aortic stenosis (AS) is the most common valvular heart disease in industrialized countries, largely driven by aging populations and the increasing prevalence of calcific degeneration of the aortic valve. Population studies report a prevalence of moderate-to-severe AS of ~2–4% among adults over 65 years and up to 10% in those older than 80 years [1,2]. Degenerative calcific AS accounts for the majority of cases, and its burden is expected to rise with aging populations. In comparison, mitral regurgitation (MR) is the second most frequent valvular heart disease (VHD), while clinically significant mitral stenosis (MS) and tricuspid disease are less common in high-income countries [3,4]. Severe AS carries a dismal prognosis once symptoms develop, with an average survival of only 2–3 years without intervention [5,6]. Early identification of high-risk patients is therefore critical for optimizing the timing of aortic valve replacement (AVR).

Importantly, AS also has a profound impact on the patients’ quality of life (QoL) due to progressive dyspnea, angina, and functional limitation. However, in individuals with advanced age, significant comorbidities, and frailty, the expected QoL benefit after valve intervention may be attenuated by limited functional reserve and competing health risks [7].

Transthoracic echocardiography (TTE) remains the cornerstone of diagnostic evaluation in AS, enabling the assessment of aortic valve area (AVA), mean transvalvular gradient, and left ventricular (LV) systolic performance [5]. While indispensable, resting echocardiographic parameters may be inconclusive in several clinically significant scenarios.

A major limitation is discordant grading, defined by an AVA ≤ 1.0 cm^2^ and a mean gradient < 40 mmHg. This pattern occurs in up to one-third of patients with severe AS and is most prevalent in the spectrum of low-flow, low-gradient (LFLG) AS, which may present either with classical LFLG, characterized by reduced LV ejection fraction (LVEF), or paradoxical LFLG, characterized by preserved LVEF and small LV cavities [8,9,10,11,12]. In such cases, resting measurements may underestimate the true valve severity and fail to distinguish between truly severe and pseudo-severe disease [13,14].

Another diagnostic challenge is asymptomatic severe AS, where patients may remain clinically silent despite advanced obstruction. Reliance on history and resting echocardiographic indices alone risks the under-recognition of subclinical LV dysfunction or maladaptive hemodynamics [15,16]. In these individuals, resting imaging cannot reliably identify those at imminent risk of symptom onset or adverse outcomes [17,18].

Collectively, these limitations underscore the need for dynamic hemodynamic assessment, most notably through stress echocardiography (SE), to complement baseline imaging. By unmasking the flow dependence of gradients, contractile reserve, exercise-induced pulmonary hypertension, and latent symptoms, stress testing and SE provide incremental diagnostic and prognostic value beyond static evaluation [5,6,19,20].

## 2. Aim of the Review

This narrative review aims to synthesize current evidence regarding the clinical role of SE in AS including its diagnostic, prognostic, and therapeutic implications as well as consensus and guideline recommendations.

## 3. Methods

A structured literature search was conducted in PubMed and Embase from January 2000 to March 2025. Search terms included ‘aortic stenosis’, stress echocardiography’, ‘exercise echocardiography’, and ‘dobutamine echocardiography’. We included randomized trials, prospective studies, meta-analyses, consensus statements, and guideline documents. Reference lists of relevant papers were screened to identify additional studies. The synthesis was organized narratively.

## 4. Principles of Stress Echocardiography in Aortic Stenosis

### 4.1. Pathophysiological Rationale in Aortic Stenosis

The hemodynamic burden imposed by AS is inherently flow-dependent. Transvalvular gradients rise disproportionately with stress-induced augmentation of stroke volume and cardiac output, whereas the calculated AVA remains relatively stable. This dissociation underscores the limitations of static assessment and highlights the need for dynamic testing to capture the functional severity of stenosis under physiological conditions [8,21]. In addition, chronic LV adaptation to sustained pressure overload—via concentric remodeling and preserved ejection fraction—may conceal impending systolic or diastolic dysfunction at rest. SE provides a unique physiological interrogation of the complex LV adaptation to aortic stenosis. By increasing preload, afterload, and myocardial oxygen demand in a controlled manner, SE can unmask subclinical contractile dysfunction (loss of contractile reserve) and reveal diastolic filling abnormalities under stress conditions [19]. Beyond ventricular performance, SE enables the evaluation of global valvulo-arterial load—an integrated measure of combined valvular and vascular afterload—and provides dynamic insight into pulmonary vascular reserve and right ventricular–pulmonary artery coupling, parameters increasingly recognized as powerful prognostic determinants in AS [5]. Collectively, these stress-induced markers extend well beyond static valve metrics and offer a comprehensive, pathophysiology-driven framework for risk stratification, optimal timing of the intervention, and a deeper understanding of disease progression in aortic stenosis.

### 4.2. Types of Stress Modalities

Exercise stress echocardiography (ESE) is considered the preferred physiological modality for the functional assessment of AS in patients with preserved LVEF, particularly when they are asymptomatic or report only equivocal symptoms. It is most commonly performed using semi-supine bicycle ergometry, which allows for continuous Doppler data acquisition during incremental workloads and thereby provides comprehensive, beat-to-beat hemodynamic information [15,17]. Although treadmill protocols with post-exercise imaging remain feasible, their diagnostic value is limited by the delay between exercise termination and image capture, which may underestimate the peak hemodynamic responses. By reproducing the physiological demands of daily activity, ESE can unmask latent disease through the provocation of exertional symptoms, the detection of abnormal blood pressure responses, the quantification of an excessive rise in mean transvalvular gradient, and the identification of exercise-induced pulmonary hypertension. Each of these exercise-derived parameters has been consistently validated as a marker of adverse prognosis in otherwise asymptomatic patients with severe AS and preserved systolic function [18,22].

In contrast, dobutamine stress echocardiography (DSE) is the test of choice for patients with LFLG AS, especially when left ventricular systolic function is reduced (LVEF < 50%). Administration of low-dose dobutamine (up to 20 µg/kg/min) increases stroke volume and transvalvular flow, enabling the differentiation between true severe AS—defined by an AVA ≤ 1.0 cm^2^ that remains fixed despite a marked rise in gradient—and pseudo-severe AS, in which the apparent severity is flow-dependent and the AVA enlarges substantially with increased flow [8,9,19]. Beyond diagnostic clarification, DSE provides critical information about contractile reserve (CR)—defined as a ≥20% increase in stroke volume—which has long been recognized as a powerful prognostic and therapeutic marker in patients with LFLG AS and reduced LVEF [23,24].

The key characteristics and indications of both modalities are summarized in Table 1.

### 4.3. Protocol Standardization

The 2016 European Association of Cardiovascular Imaging (EACVI) and the American Society of Echocardiography (ASE) consensus provided the first unified framework for SE in non-ischemic heart disease, incorporating its role in AS [19]. Subsequently, the Stress Echo 2020 document advanced a multiparametric ABCDE protocol, integrating:

**A**: Regional wall motion;

**B**: Pulmonary congestion (B-lines);

**C**: Contractile and coronary flow reserve;

**D**: Diastolic reserve;

**E**: Exercise-induced arrhythmias [20].

For aortic stenosis, further refinement has been introduced with the ABCDEG protocol, in which **G** denotes the dynamic assessment of transaortic gradients, ensuring systematic acquisition and interpretation of stress-induced valve hemodynamics [25].

### 4.4. Safety Considerations and Contraindications

Both modalities have been demonstrated to be safe in specialized centers, with low complication rates, below 1% per year, even among patients with advanced AS traditionally perceived as high-risk [18,26]. Contraindications include hemodynamic instability, very severe symptomatic AS, and the inability to perform or tolerate the protocol. International consensus documents emphasize rigorous patient selection, close monitoring, and expertise in test execution as prerequisites for safe deployment [5,19,20].

## 5. Exercise Stress Echocardiography in Asymptomatic Severe Aortic Stenosis

### 5.1. Rationale for Exercise Testing

A considerable subset of patients with severe AS remain ostensibly asymptomatic despite advanced valvular obstruction. Symptom-based assessment is inherently limited, as many patients subconsciously curtail physical activity to avoid exertional discomfort, thereby masking clinically significant limitation. This adaptive behavior renders reliance on history alone unreliable for risk stratification.

In this context, ESE serves as an essential investigative modality, providing an objective quantification of functional capacity and the ability to unmask latent symptoms. Moreover, it permits the characterization of the dynamic hemodynamic burden of AS under physiological stress conditions, thereby bridging the gap between resting echocardiographic parameters and true disease severity in daily life [15,17,22].

### 5.2. Evidence Base and Prognostic Markers During ESE

The prognostic utility of ESE in asymptomatic severe AS is supported by a robust body of evidence. In a landmark prospective investigation, “Lancellotti et al., 2005” demonstrated that an exercise-induced rise in mean transvalvular gradient conveyed incremental prognostic information beyond resting echocardiographic indices, thereby refining risk stratification [15]. This observation was corroborated by “Maréchaux et al., 2010”, who showed that abnormal hemodynamic responses during ESE identified asymptomatic patients at elevated risk for adverse cardiovascular outcomes [27]. Extending these insights, “Lancellotti et al., 2012” highlighted the occurrence of exercise-induced pulmonary hypertension as a particularly powerful predictor of symptom development and cardiovascular mortality [16].

The accumulated evidence was subsequently synthesized in a state-of-the-art review by “Magne et al., 2014”, which concluded that ESE represents a valuable adjunct in the evaluation of asymptomatic severe AS, particularly in patients with preserved LVEF [17]. More contemporary data from “Abergel et al., 2025” and “Rassi et al., 2013” have reinforced these findings, emphasizing both the prognostic significance and safety profile of ESE in this population [18,26]. Finally, in the most recent contribution, “Abergel et al., 2025” demonstrated that serial ESE provides dynamic surveillance of disease progression, with repeated hemodynamic testing enhancing prognostic accuracy and improving the timing of intervention [18]. Collectively, these investigations demonstrate that ESE not only identifies high-risk patients through hemodynamic and symptomatic responses, but also confirms the feasibility and safety of this approach in specialized centers. Table 2 summarizes the principal parameters derived from ESE in asymptomatic severe AS.

### 5.3. Clinical Decision-Making

The results of ESE have a direct and actionable impact on the management of asymptomatic severe AS. The detection of adverse hemodynamic responses—including an excessive rise in mean transvalvular gradient, exercise-induced pulmonary hypertension, an abnormal blood pressure response, or the provocation of symptoms—has consistently been associated with poor outcomes and serves as a compelling argument for earlier consideration of AVR, even when classical guideline-defined triggers (symptoms at rest, depressed LV ejection fraction, or very severe stenosis) are absent [15,16,17,18,27]. This paradigm has been explicitly endorsed in both the 2021 European Society of Cardiology (ESC) and European Association for Cardio-Thoracic Surgery (EACTS) Guidelines and the 2020 American College of Cardiology and American Heart Association ACC/AHA Guidelines, which recognize the role of ESE in refining the timing of intervention in high-risk but ostensibly asymptomatic patients [5,6].

Conversely, the identification of a normal ESE profile, characterized by preserved exercise tolerance, stable gradients, appropriate blood pressure augmentation, and the absence of pulmonary hypertension, delineates a subgroup with a relatively low short-term event risk. In such patients, the strategy of watchful waiting with careful clinical and echocardiographic surveillance is both safe and appropriate [26,28,29]. More recently, longitudinal investigations have further demonstrated that serial ESE testing may enhance dynamic risk stratification and optimize the timing of intervention [18].

Collectively, this body of evidence underscores the central role of ESE as a gatekeeper in the nuanced decision-making process of asymptomatic severe AS, balancing the avoidance of premature intervention against the risk of delayed surgery and irreversible myocardial decompensation.

## 6. Dobutamine Stress Echocardiography in Low-Flow, Low-Gradient Aortic Stenosis

### 6.1. The Clinical Problem of Low-Flow, Low-Gradient Aortic Stenosis

LFLG aortic stenosis represents one of the most diagnostically and therapeutically challenging phenotypes within the spectrum of AS. It is characterized by a discordance between an anatomically severe valve lesion—typically evidenced by heavy calcification, a calculated AVA ≤ 1.0 cm^2^, and unexpectedly low transvalvular gradients at rest (<40 mmHg). This discordance complicates disease grading and frequently obscures the true severity of obstruction [9,10].

Two principal forms are recognized. Classical LFLG AS occurs in the setting of reduced LVEF, where the stroke volume is diminished as a consequence of impaired contractility. In contrast, paradoxical LFLG AS develops despite preserved LVEF and is typically associated with concentric left ventricular remodeling, small ventricular cavities, impaired diastolic filling, and reduced longitudinal strain [9,30,31].

In both entities, reliance on resting echocardiographic indices alone risks the misclassification of severity and inappropriate deferral of intervention. Differentiating true severe AS (fixed obstruction with poor flow) from pseudo-severe AS (apparent severity driven by reduced forward stroke volume rather than fixed valve narrowing) is therefore of paramount importance, as management pathways and prognosis diverge substantially between these groups [13,14,23].

Two principal interpretative criteria are applied. First, the assessment of CR, conventionally defined as a ≥20% increase in stroke volume, provides insight into left ventricular recruitability. The presence of CR is strongly associated with improved operative outcomes and survival following surgical or transcatheter aortic valve replacement, whereas its absence portends a poorer prognosis [13,24,32].

Second, the analysis of valve hemodynamics under flow augmentation distinguishes between true and pseudo-severe AS. A diagnosis of true severe AS is supported when the mean transvalvular gradient rises to ≥40 mmHg while the AVA remains ≤1.0 cm^2^, indicating fixed obstruction. Conversely, a marked increase in AVA to >1.0 cm^2^ with only modest gradient augmentation suggests pseudo-severe AS, in which reduced stroke volume rather than fixed stenosis accounts for the resting hemodynamic profile [13,14,23].

Through this dual assessment of myocardial reserve and valve anatomy–hemodynamic interplay, DSE provides critical information that directly informs therapeutic decision-making in the complex subset of LFLG AS.

### 6.2. Projected Aortic Valve Area

A recognized limitation of conventional DSE is that a substantial proportion of patients with LFLG AS fail to achieve adequate augmentation of stroke volume or flow, rendering standard diagnostic criteria inconclusive. To overcome this limitation, Blais et al. (2006) introduced the concept of the projected aortic valve area (AVAproj), which extrapolates the AVA to a standardized flow rate of 250 mL/s [14]. This approach minimizes the dependence on the degree of actual flow achieved during DSE and provides a more robust estimate of the “true” severity of valve obstruction.

The prognostic utility of AVAproj was subsequently confirmed in the TOPAS multicenter study (Clavel et al., 2010 [33]), which demonstrated that AVAproj more accurately predicted operative mortality and long-term outcomes than conventional DSE-derived parameters alone [25]. Additional studies have reinforced these findings, highlighting the value of AVAproj, particularly in patients with absent contractile reserve, in whom traditional DSE criteria frequently fail [23,32].

More recently, integration of AVAproj into comprehensive diagnostic algorithms has been endorsed in expert consensus documents and position statements from the European Association of Cardiovascular Imaging (EACVI) and the ASE and acknowledged in the 2021 ESC/EACTS and 2020 ACC/AHA Guidelines [5,6,19,20]. These documents recognize AVAproj as a valuable adjunct for distinguishing true from pseudo-severe AS, refining operative risk stratification and improving patient selection for surgical and transcatheter valve interventions.

Table 3 outlines the main diagnostic approaches using DSE to distinguish true severe from pseudo-severe LFLG AS. Both standard DSE criteria and AVAproj have been validated, each with distinct strengths and limitations that guide their clinical application.

### 6.3. Prognostic Implications

The prognostic significance of findings obtained during DSE has been extensively investigated in the setting of LFLG AS.

In patients with classical LFLG AS and reduced LVEF, the presence or absence of CR—defined as a ≥20% increase in stroke volume during low-dose dobutamine infusion—was historically regarded as a critical determinant of operative risk. Early surgical series demonstrated that the absence of CR was associated with operative mortality rates approaching 30–50%, reflecting both advanced myocardial dysfunction and limited capacity to tolerate surgical stress [13,24]. However, more contemporary evidence from the transcatheter aortic valve replacement (TAVR) era indicates that the prognostic weight of CR has diminished. Modern transcatheter and surgical interventions confer a substantial survival benefit, even in patients without demonstrable CR, suggesting that the absence of flow reserve should no longer be viewed as an absolute contraindication to intervention [23].

The distinction between true severe and pseudo-severe AS remains fundamental. Patients confirmed to have true severe stenosis derive a clear survival advantage from timely valve replacement. In contrast, those with pseudo-severe AS do not benefit from intervention and are best managed conservatively with optimized medical therapy [23,34]. This differentiation, uniquely provided by DSE, therefore serves as a cornerstone of clinical decision-making in this complex subgroup.

Finally, in the setting of paradoxical LFLG AS with preserved LVEF, DSE has also proven valuable. “Clavel et al., 2013” demonstrated that stress-induced hemodynamic responses allow for the stratification of patients in whom forward flow is reduced due to concentric remodeling, small ventricular cavities, and impaired diastolic filling. In these cases, the assessment of flow reserve and gradient behavior contributes to the improved characterization of disease severity and risk stratification, extending the applicability of DSE beyond patients with impaired systolic function [32].

Table 4 summarizes the key prognostic markers obtained from DSE in LFLG AS. These markers—including CR, true versus pseudo-severe AS, and paradoxical LFLG patterns—provide critical diagnostic and therapeutic guidance, directly influencing patient selection and timing for valve intervention.

### 6.4. Recent Refinements and Contemporary Insights

Several methodological and conceptual refinements have advanced the application of DSE in the evaluation of LFLG AS.

First, the conventional reliance on stroke volume index as a surrogate for flow augmentation has been challenged. “Kadem et al., 2006” demonstrated that the stress transaortic flow rate provides a more direct and physiologically robust measure of forward flow, thereby enhancing the diagnostic accuracy and reducing misclassification in DSE interpretation [35].

Second, contemporary multicenter investigations have consolidated the evidence base. In a large-scale analysis, “Mogensen et al., 2024” JASE study confirmed the safety and diagnostic utility of DSE across a broad range of ejection fractions [11]. Importantly, this work also refined diagnostic cut-offs for both CR and the AVAproj, thereby harmonizing DSE criteria with modern practice standards [11].

Finally, the role of multimodality imaging has become increasingly prominent when DSE results remain inconclusive. Both the 2021 ESC/EACTS Guidelines and the 2020 ACC/AHA Guidelines endorse the use of computed tomography calcium scoring (CT-CAC) as a complementary diagnostic tool, particularly in patients with paradoxical LFLG AS, where calcification burden offers incremental value for confirming stenosis severity [5,6].

Collectively, these refinements underscore the ongoing evolution of DSE from a niche diagnostic tool into a central component of the multimodality assessment strategy for complex AS phenotypes.

Table 5 highlights recent refinements in the assessment of LFLG AS, focusing on innovations in DSE and its integration with multimodality imaging. These advances aim to improve diagnostic accuracy, standardize interpretation, and enhance risk stratification in complex patient subsets.

### 6.5. Comparative Assessment of Dobutamine Stress Echocardiography and Computed Tomography Calcium Scoring in Low-Flow, Low-Gradient Aortic Stenosis

Accurate grading of AS is challenging in LFLG AS including paradoxical LFLG AS with preserved LVEF. Standard echocardiography may underestimate severity when flow is reduced, leading to discordant findings. ESC/EACTS Guidelines (2021) recommend a multimodality approach, with DSE and CT-CAC as key complementary tools [5,38].

DSE evaluates AS severity under pharmacologic flow augmentation. True severe AS is defined by a mean gradient ≥ 40 mmHg and/or AVA ≤ 1.0 cm^2^ with ≥20% stroke volume increase, while pseudo-severe AS shows AVA > 1.0 cm^2^ without gradient rise [12]. Additionally, DSE provides information on contractile reserve (≥20% increase in stroke volume), a key prognostic marker associated with lower operative risk and improved long-term outcomes [24,39].

CT-CAC offers a flow-independent, highly reproducible, and operator-independent method to quantify the anatomical burden of aortic valve calcification. Using the Agatston scoring system, CT-CAC overcomes key limitations of DSE—particularly when contractile reserve is absent, when patients have arrhythmias, or when echocardiographic windows are suboptimal [36]. Sex-specific thresholds have been validated for the diagnosis of severe AS: ≥2000 Agatston units (AU) in men and ≥1200 AU in women.

Values above these cut-offs strongly support the diagnosis of true severe AS and predict adverse clinical outcomes including mortality and rapid disease progression [36,37]. Unlike DSE, CT-CAC is unaffected by loading conditions or hemodynamic variability, making it especially useful in frail or unstable patients.

DSE and CT-CAC should be regarded as complementary modalities rather than competing techniques. DSE remains the first-line functional test for distinguishing true from pseudo-severe AS in LFLG settings, as it provides dynamic insight into both valve hemodynamics and left ventricular contractile reserve. In contrast, CT-CAC offers a robust, anatomy-based criterion that is particularly valuable when DSE is inconclusive or infeasible.

According to the 2021 ESC/EACTS Guidelines, CT-CAC holds a Class I recommendation for confirming severe AS in patients with discordant echocardiographic findings or when DSE cannot provide definitive results [5,38]. An integrated multimodality approach—starting with DSE and complemented by CT-CAC when necessary—enhances diagnostic accuracy, minimizes the risk of misclassification, and guides the optimal timing of aortic valve intervention.

Table 6 below summarizes the principal differences, strengths, limitations, and guideline recommendations for each technique to assist in clinical decision-making.

#### Limitations of Stress Echocardiography and the Role of CT-CAC

While SE provides important functional and prognostic information in AS, several limitations may affect its reliability and reproducibility. SE is inherently operator dependent, requiring expertise in image acquisition and interpretation, which can lead to variability across centers and examiners [13]. Diagnostic accuracy may also be compromised in patients with suboptimal acoustic windows, such as those with obesity, chronic obstructive pulmonary disease (COPD), or chest wall deformities, where visualization of the left ventricle and aortic valve can be challenging [41].

In contrast, CT-CAC scoring is largely operator independent, with standardized acquisition and post-processing protocols, and shows high interobserver reproducibility [36,37]. These attributes make CT-CAC particularly valuable when the SE results are technically limited, non-diagnostic, or discordant, reinforcing the complementary role of CT-CAC in the multimodality assessment of AS.

## 7. Hemodynamic and Functional Parameters During Stress Echocardiography

SE in AS provides far more than a binary assessment of stenosis severity. By interrogating multiple hemodynamic and functional parameters during stress, clinicians can capture the dynamic interplay between the valve, ventricle, and pulmonary circulation. These parameters, some of which we have already discussed, carry incremental diagnostic and prognostic value beyond the standard valve area and mean gradient.

### 7.1. Left Ventricular Systolic Reserve

Assessment of CR is a cornerstone of DSE, particularly in classical LFLG AS with reduced ejection fraction. As we have mentioned, a ≥20% increase in stroke volume indicates preserved myocardial recruitability, which is typically associated with lower operative mortality [12,32]. While the absence of CR once portended prohibitive surgical risk, more recent studies have demonstrated that contemporary AVR (especially TAVR) confers meaningful survival even in these patients [31,32].

### 7.2. Left Ventricular Diastolic Function

ESE offers insights into diastolic reserve. As we have discussed, patients with advanced AS frequently exhibit impaired LV relaxation and increased filling pressures that are not evident at rest. Stress-induced increases in the E/e′ ratio or left atrial pressure surrogates correlate with exertional dyspnea and predict earlier symptom development [17,42].

### 7.3. Transaortic Flow and Gradients

Dynamic evaluation of transaortic gradients during stress testing constitutes a cornerstone of functional assessment in AS. The flow rate, calculated as stroke volume ÷ LV ejection time under stress, provides a more physiologic and reproducible assessment of flow augmentation; it improves diagnostic accuracy compared with the stroke volume index. Both ESE and DSE values of transaortic flow and gradients are especially valuable in inconclusive cases of LFLG AS [35].

### 7.4. Myocardial Deformation Imaging

The incorporation of strain imaging into SE protocols has provided a more sensitive means of detecting subclinical LV dysfunction in AS. Global longitudinal strain (GLS) offers incremental prognostic information beyond conventional measures of LVEF. During exercise or pharmacologic stress, the failure of GLS to augment appropriately reflects limited myocardial contractile reserve, a phenomenon associated with impaired outcomes even in patients with preserved LVEF [20,41].

Several studies have highlighted the prognostic utility of strain-derived indices in AS. “Vollema et al., 2018” and “Zhu et al., 2020” demonstrated that impaired GLS predicts symptom development and adverse cardiovascular events in asymptomatic severe AS [40,43]. Similarly, “Dahl et al., 2012” reported that reduced stress GLS correlates with adverse remodeling and higher mortality risk, while “Stens et al., 2023” confirmed its value in refining risk stratification in patients undergoing TAVR [44,45]. Beyond GLS, regional strain patterns have also been linked to outcomes, with “Levy-Neuman et al., 2019” showing that impaired basal strain under stress is particularly prognostic [46].

In line with these data, expert consensus statements have begun to endorse the integration of myocardial deformation imaging into advanced SE protocols as part of a multiparametric approach to assess CR, hemodynamics, and global LV function [19,42].

### 7.5. Valvulo-Arterial Impedance and Global Afterload

The hemodynamic burden imposed on the LV in AS is determined not only by the degree of valvular obstruction, but also by the arterial system into which the ventricle ejects. The concept of valvulo-arterial impedance (Zva), introduced by “Dahl et al. 2012”, integrates transvalvular pressure gradients with systemic vascular resistance, thereby providing a comprehensive measure of global LV afterload [44]. Elevated Zva has been consistently associated with impaired LV systolic performance, reduced exercise tolerance, and increased mortality in patients with AS [47,48,49].

SE has emerged as a valuable modality to assess the dynamic behavior of Zva. Exercise or pharmacologic stress may reveal disproportionate increases in Zva, reflecting the inability of the arterial system to accommodate increased cardiac output. Such stress-induced augmentation of global afterload has been linked to accelerated symptom onset, adverse remodeling, and poorer prognosis [9,48,50].

More recent work has emphasized the prognostic and therapeutic relevance of this integrative parameter. “Minners et al., 2010” demonstrated that Zva adds incremental prognostic value over conventional indices of AS severity, particularly in patients with paradoxical low-flow states [51]. Similarly, “Magne et al., 2014” showed that elevated Zva identifies asymptomatic patients at higher risk of progression and adverse outcomes, supporting its use in risk stratification [17]. Finally, consensus recommendations increasingly recognize Zva as a complementary parameter that contextualizes the valvular lesion within the broader framework of ventriculo-arterial coupling [5,19,20].

### 7.6. Concept of Volume–Flow (V–Q) Discordance

Recent findings from the ESC Congress 2025, simultaneously published in JACC: Asia, introduced the concept of volume–flow (V–Q) discordance as a novel prognostic marker in severe AS. In a cohort of 291 patients (≥65 years) undergoing TAVR with a median follow-up of 3 years, approximately 29% exhibited V–Q discordance—defined by discrepancies between the stroke volume index (SVi) and the transaortic flow rate (TFR). Patients with low V–Q discordance (SVi < 35 mL/m^2^ with TFR > 210 mL/s) demonstrated significantly better 3-year survival (86.0% vs. 73.8%, *p* = 0.03) compared with concordant profiles. Importantly, V–Q discordance provided superior prognostic discrimination compared with either the SVi or TFR alone. The study suggests that incorporating this parameter could refine risk stratification, prognostic assessment, and clinical decision-making in severe AS, particularly in patients with low-flow states. Future research should focus on validating standardized cut-offs and integrating flow rate analysis into multimodality imaging algorithms [52].

Table 7 summarizes the key functional indices assessed during stress testing, their preferred modality, and their clinical relevance for risk stratification and management.

## 8. Guideline Recommendations and Consensus Statements

International consensus documents and guidelines consistently endorse SE for AS. The ESC/EACTS Guidelines recommend ESE for risk stratification in asymptomatic severe AS (Class IIa) and DSE in classical LFLG AS (Class I) [5,55]. The 2020 ACC/AHA Guidelines provide similar recommendations, also supporting integration with CT-CAC when results are inconclusive [6]. The Stress Echo 2020 document introduced a multiparametric ABCDE protocol, later refined as ABCDEG, to systematically assess transaortic gradients [27].

Together, these consensus statements and guideline recommendations position SE as a versatile, multiparametric imaging modality, uniquely suited to bridge the gap between anatomical severity and functional impact in aortic stenosis, with direct implications for diagnosis, prognosis, and therapeutic guidance.

Table 8 provides an overview of the evolution of SE in AS across expert consensus documents and international guidelines. These publications have progressively expanded the role of SE—from early recommendations in asymptomatic severe and LFLG AS to its integration within multiparametric and multimodality frameworks—culminating in formal guidelines.

## 9. Clinical Scenario and Utility for Using SE in AS

Both societies, ESC/EACTS and ACC/AHA, recognize the central role of DSE in the evaluation of classical LFLG AS with reduced EF, where it provides critical information to distinguish true severe from pseudo-severe stenosis [5,6]. This constitutes an area of strong consensus, although minor differences exist in the grading of the level of evidence.

In contrast, recommendations for ESE in asymptomatic severe AS show divergence: the ESC/EACTS Guidelines grant a stronger recommendation (Class I, LOE C), whereas the ACC/AHA Guidelines consider it only reasonable (Class IIa, LOE B-NR). Similarly, the ESC/EACTS Guidelines provide broader guidance on the integration of SE with multimodality imaging—including CT-CAC—in paradoxical LFLG AS, whereas the ACC/AHA Guidelines remain more conservative [5,6]. These differences highlight the evolving role of SE across regions and the need for harmonization in future updates.

**Figure 1:**
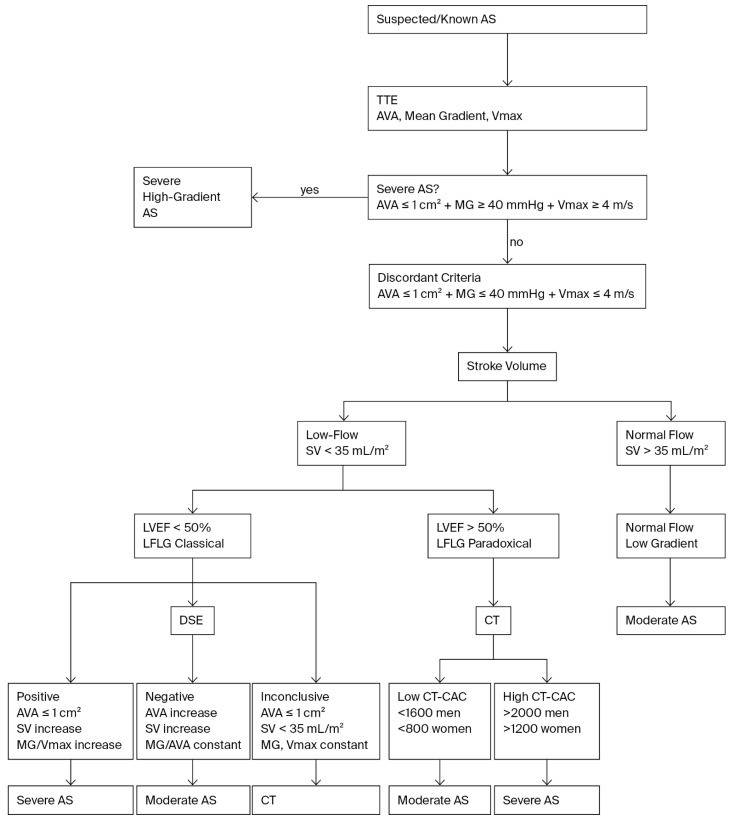
Diagnostic flowchart for the assessment of aortic stenosis according to the latest ESC/EACTS Guidelines. Abbreviations: AS—aortic stenosis; AVA—aortic valve area; MG—mean gradient; Vmax—maximum jet velocity; SV—stroke volume; LVEF—left ventricular ejection fraction; LFLG—low-flow, low-gradient; DSE—dobutamine stress echocardiography; CT—computed tomography; CT-CAC—computed tomography calcium scoring; ESC—European Society of Cardiology.

A diagnostic flowchart outlining the evaluation pathway according to the most recent ESC/EACTS Guidelines is shown in Figure 1.

## 10. Knowledge Gaps and Future Directions

### 10.1. Integration with Multimodality Imaging

Although SE offers unique physiological information regarding valvular and myocardial function, its diagnostic accuracy can be constrained in patients with suboptimal acoustic windows or insufficient hemodynamic augmentation. Current guidelines support the integration of CT-CAC as an adjunctive tool for AS severity when DSE yields inconclusive results, particularly in the setting of paradoxical LFLG AS [5,6]. In parallel, cardiac magnetic resonance (CMR) provides incremental value by enabling tissue characterization, specifically the detection and quantification of myocardial fibrosis, thereby linking contractile reserve assessed by SE to underlying structural remodeling [41,48]. Emerging evidence suggests that a multimodality approach—integrating SE with CT-CAC and CMR—may establish a comprehensive imaging paradigm that captures both the hemodynamic and structural dimensions of valvular heart disease.

### 10.2. Advanced Echocardiographic Technologies

Several novel echocardiographic modalities are being integrated into SE protocols to enhance diagnostic precision in AS. Myocardial strain imaging enables sensitive quantification of LV contractile reserve, with impaired augmentation of GLS during stress emerging as a predictor of adverse outcomes even in the presence of preserved LVEF [41,43]. Three-dimensional (3D) echocardiography has the potential to improve the accuracy of stroke volume and AVA measurements under stress conditions, mitigating interobserver variability compared with conventional two-dimensional approaches [27,48]. In addition, the use of contrast echocardiography facilitates a more reliable delineation of endocardial borders at high workloads, thereby enhancing the feasibility and accuracy of quantitative assessment.

### 10.3. Artificial Intelligence and Automated Analysis

The application of machine learning and artificial intelligence (AI) to SE is an emerging frontier. Automated quantification of Doppler gradients, LV volumes, and strain parameters could standardize interpretation, reduce observer variability, and facilitate multiparametric risk stratification in real-time. Early proof-of-concept studies in echocardiography suggest that AI-driven SE interpretation could significantly improve reproducibility, particularly in multicenter trials [21,58].

### 10.4. Role in the Transcatheter Era

The progressive expansion of TAVR intermediate- and lower-risk populations has intensified the need for refined tools to optimize the timing of intervention and patient selection [59,60,61]. In this evolving therapeutic landscape, SE holds considerable potential as both a diagnostic and prognostic adjunct. First, in patients with borderline or discordant AS severity, SE—whether through exercise protocols or dobutamine stress testing—can unmask latent hemodynamic compromise, thereby clarifying disease severity and strengthening indications for intervention. Second, beyond its preprocedural role, SE may contribute to post-TAVR prognostication [52]. Stress testing can detect residual pulmonary hypertension, delineate abnormal afterload responses, and assess LV contractile reserve under dynamic conditions, all of which carry important implications for functional recovery and long-term outcomes.

Finally, the concept of dynamic surveillance is emerging, with recent evidence suggesting that serial SE assessments provide incremental prognostic information and may guide the optimal timing of intervention in asymptomatic patients [26]. Such an approach may be particularly relevant in younger or lower-risk populations, where balancing the risks of premature intervention against the consequences of delayed treatment remains a key clinical challenge.

### 10.5. Standardization and Broader Adoption

Although accumulating evidence supports the diagnostic and prognostic value of SE in AS, its use remains limited compared with its established role in ischemia testing. Broader implementation will depend on three key developments.

First, protocol harmonization is required to enhance reproducibility and comparability across centers. The recently proposed ABCDEG framework, building on the multiparametric Stress Echo 2020 protocol, provides a structured approach tailored to valvular disease by integrating the systematic evaluation of stress-induced transvalvular gradients with conventional functional indices [20,42].

Second, the expansion of training and accreditation pathways is essential to strengthen expertise in the acquisition, interpretation, and clinical integration of valvular SE. Structured educational models—like those long established for ischemia testing—will be pivotal in fostering uniformity and clinician confidence.

Finally, large-scale, prospective multicenter studies are needed to validate clinically relevant thresholds for prognostic markers including the exercise-induced rise in mean gradient, cut-offs for stress-induced pulmonary hypertension, and stress-related changes in GLS. Demonstrating the incremental impact of these parameters on hard clinical outcomes will be critical to advancing SE from a specialized investigation to a mainstream component of AS evaluation and management.

### 10.6. Barriers to the Widespread Use of Stress Echocardiography in Routine Clinical Practice

Despite its established diagnostic and prognostic value in aortic stenosis (AS), the routine use of SE—especially DSE—remains limited by several factors.

First, SE is highly operator dependent and requires advanced training in image acquisition, hemodynamic interpretation, and stress protocol management, leading to inter-center variability and reduced reproducibility compared with more standardized imaging modalities such as CT-CAC [5,20].

Second, technical limitations such as suboptimal acoustic windows in patients with obesity, COPD, or previous thoracic surgery may compromise image quality and diagnostic reliability [8].

Third, logistical challenges, including the need for dedicated echocardiography equipment, pharmacologic stress agents, continuous monitoring, and trained staff, can limit availability, particularly in non-tertiary centers.

Additionally, the lack of standardized reporting templates and cut-off values across laboratories as well as the perception of increased complexity and time consumption may discourage routine use [5].

Finally, while guideline support exists, awareness and confidence among clinicians in interpreting SE results in the context of low-flow, low-gradient AS may still be insufficient compared with the simpler, quantitative nature of CT-CAC.

Together, these barriers highlight the need for training standardization, streamlined protocols, and a wider dissemination of guideline-based diagnostic algorithms to promote a more consistent integration of SE into everyday clinical practice.

## 11. Conclusions

Stress echocardiography has become an essential adjunct in the evaluation of AS, overcoming key limitations of resting echocardiography. Its role is particularly established in two domains: ESE, which unmasks latent symptoms and hemodynamic abnormalities in asymptomatic severe AS, and DSE, which clarifies stenosis severity in LFLG AS and provides prognostic insights through the assessment of contractile reserve and projected AVA.

Supported by international consensus and guideline recommendations, SE now holds Class I–IIa indications in selected scenarios, providing incremental diagnostic and prognostic value and complementing multimodality imaging such as CT-CAC. Future directions include standardized multiparametric protocols (ABCDEG), the integration of deformation and 3D imaging, and the application of artificial intelligence to enhance reproducibility and quantification.

In the era of transcatheter therapies, SE is poised to refine patient selection, optimize intervention timing, and support dynamic surveillance, affirming its place as a cornerstone of precision medicine in AS.

In summary, stress echocardiography is no longer a niche investigation but a cornerstone of precision medicine in aortic stenosis, bridging anatomy and physiology to guide individualized patient care.

The key takeaways are as follows:

SE overcomes the limitations of resting echo, refining diagnosis and risk stratification in AS.

ESE unmasks latent symptoms and hemodynamic abnormalities in asymptomatic severe AS and is supported by guidelines for early risk stratification.

DSE differentiates true vs. pseudo-severe LFLG AS and provides prognostic insights via CR and projected AVA.

Integration with CT-CAC, CMR, and strain imaging enhances diagnostic accuracy and prognostication.

Future directions include standardized multiparametric protocols (ABCDEG), AI-driven analysis, and broader adoption in the TAVR era.

## Figures and Tables

**Table 1:** Comparative use of exercise vs. dobutamine stress echocardiography in aortic stenosis.

Feature	Exercise Stress Echocardiography	Dobutamine Stress Echocardiography
Primary patient population	Asymptomatic or equivocal-symptom severe AS with preserved LVEF	LFLG AS with reduced LVEF (<50%)
Main purpose	Risk stratification and unmasking of latent symptoms Functional hemodynamic assessment under physiological load	Differentiates true severe vs. pseudo-severe AS in LFLG states Evaluates CR
Stress modality	Semi-supine bicycle ergometry (preferred) Treadmill with post-exercise imaging (less ideal)	Low dose dobutamine infusion (≤20 μg/kg/min)
Key parameters assessed	Symptom provocation (angina, dyspnea, and syncope) Abnormal blood pressure response Change in mean transvalvular gradient with exercise Exercise-induced pulmonary hypertension	Change in stroke volume (CR: ≥20% rise) Flow-induced changes in mean gradient AVA stability (true severe: ≤1.0 cm^2^ despite ↑ flow; pseudo-severe: AVA enlarges)

Abbreviations: AS = aortic stenosis; AVA = aortic valve area; CR = contractile reserve; LFLG AS = low-flow, low-gradient aortic stenosis; LVEF = left ventricular ejection fraction; LV = left ventricle. ↑ increase.

**Table 2:** Integrated evidence on exercise stress echocardiography in asymptomatic severe aortic stenosis: parameters, thresholds, clinical significance, and key supporting studies.

Parameter	Definition/Threshold	Clinical Significance	Impact
Rise in mean transvalvular gradient [14,27]	Exercise-induced increase ≥18–20 mmHg	Associated with accelerated symptom development and adverse prognosis	First demonstration of incremental prognostic value of ESE beyond resting indices; validated ESE as a stratification tool in asymptomatic patients
Exercise-induced pulmonary hypertension [16]	Pulmonary artery systolic pressure >60 mmHg	Reflects impaired LV–pulmonary vascular coupling; strong predictor of mortality and AVR	Introduced pulmonary hypertension as a novel prognostic marker
Blood pressure response [17]	Abnormal or blunted rise in systolic pressure during exercise	Indicates limited LV contractile reserve; associated with worse outcomes	Validated prognostic relevance; established ESE as valuable adjunct in severe AS with preserved EF
Symptom provocation during ESE [18,22]	Development of exertional dyspnea, angina, or presyncope	Provides unequivocal evidence of decompensation despite stable resting echocardiography	Strong clinical indicator; supported broader application in guidelines
Safety/feasibility of ESE [18,26]	—	Demonstrates that ESE is a practical and generally safe tool in severe AS	Confirmed feasibility and safety in clinical practice
Serial/longitudinal ESE value [18]	—	Useful for disease monitoring and timing of AVR intervention	Highlighted role of repeated ESE in longitudinal surveillance

Abbreviations: AS = aortic stenosis; AVR = aortic valve replacement; EF = ejection fraction; ESE = exercise stress echocardiography; LV = left ventricle.

**Table 3:** Diagnostic approaches for differentiating true severe vs. pseudo-severe low-flow, low-gradient aortic stenosis using dobutamine stress echocardiography.

Approach	Definition/Method	Strengths	Limitations
Standard DSE criteria [13,14,23,24,32]	Low dose dobutamine infusion up to 20 μg/kg/min; interpretation based on the following: True severe AS: mean gradient ≥40 mmHg with AVA ≤ 1.0 cm^2^ Pseudo-severe AS: AVA increases >1.0 cm^2^ with minimal gradient rise	Widely validated in classical LFLG AS Provides information on valve hemodynamics and contractile reserve Strong prognostic implications when criteria are conclusive	30–40% of patients fail to achieve sufficient flow augmentation Inconclusive in absence of contractile reserve Risk of underestimating severity in borderline cases
Projected AVA (AVAproj) [5,6,12,14,19,32,33]	Mathematical extrapolation of AVA to a standardized flow of 250 mL/s, derived from slope of AVA–flow relationship during DSE	Overcomes limitations of insufficient flow increase Provides standardized comparison across patients Superior predictor of operative mortality and long-term outcomes Valuable in patients without contractile reserve	Requires accurate Doppler and flow measurement Not universally implemented in routine practice Limited availability in some echo systems

Abbreviations: AS = aortic stenosis; AVA = aortic valve area; AVAproj = projected aortic valve area; DSE = dobutamine stress echocardiography; LFLG = low-flow, low-gradient.

**Table 4:** Prognostic markers derived from dobutamine stress echocardiography in low-flow, low-gradient aortic stenosis: clinical significance and therapeutic implications.

Marker	Definition/Finding	Prognostic Significance	Therapeutic Implications
Contractile reserve (CR) [13,23,24]	≥20% increase in stroke volume during low dose dobutamine	Historically, absence of CR predicted high operative mortality (30–50%) in surgical AVR	Presence of CR = better surgical/TAVR outcomes; absence no longer absolute contraindication with contemporary AVR
True severe AS [13,14,23]	Mean gradient ≥40 mmHg with AVA ≤1.0 cm^2^ during flow augmentation	Identifies patients with fixed obstruction at high risk of adverse outcomes if untreated	AVR (SAVR or TAVR) confers substantial survival benefit
Pseudo-severe AS	AVA increases >1.0 cm^2^ with minimal gradient rise during DSE	Reflects flow limitation rather than fixed obstruction; not associated with improved survival after AVR	Conservative management preferred; avoids unnecessary intervention
Paradoxical LFLG AS [31,32]	Preserved EF, small LV cavity, impaired filling; gradient remains low despite flow	DSE refines risk stratification, identifying those with severe obstruction despite preserved EF	Guides selection for AVR vs. watchful waiting

Abbreviations: AS = aortic stenosis; AVR = aortic valve replacement; SAVR = surgical AVR; TAVR = transcatheter AVR; AVA = aortic valve area; EF = ejection fraction; LV = left ventricle; LFLG = low-flow, low-gradient; DSE = dobutamine stress echocardiography; CR = contractile reserve.

**Table 5:** Recent refinements in the assessment of LFLG AS using dobutamine stress echocardiography and multimodality imaging.

Refinement/Insight	Definition/Key Proposal	Clinical Impact
Flow rate vs. stroke volume index [36]	Use of stress transaortic flow rate instead of stroke volume index as the preferred marker of flow augmentation	Improves diagnostic accuracy by directly reflecting transvalvular hemodynamics; reduces risk of misclassification
Contemporary multicenter evidence [37]	Large-scale registry analysis of DSE across a spectrum of LVEF; refined diagnostic cut-offs for contractile reserve and AVAproj	Confirms safety of DSE; standardizes interpretation criteria; supports use in both classical and paradoxical LFLG AS
Integration with multimodality imaging [5,6]	Incorporation of CT-CAC when DSE is inconclusive, particularly in paradoxical LFLG AS	Enhances diagnostic certainty; complements DSE by confirming anatomical severity of stenosis

Abbreviations: AS = aortic stenosis; AVR = aortic valve replacement; LVEF = left ventricular ejection fraction; AVAproj = projected aortic valve area; LFLG = low-flow, low-gradient; DSE = dobutamine stress echocardiography; CT-CAC = computed tomography calcium scoring.

**Table 6:** Comparative features of dobutamine stress echocardiography and computed tomography calcium scoring (CT-CAC) for the diagnosis of true severe aortic stenosis in low-flow, low-gradient states.

Feature	Dobutamine Stress Echocardiography	Computed Tomography Calcium Scoring
Principle [13,36,37]	Flow-dependent, functional assessment of AS severity under pharmacologic augmentation of stroke volume	Flow-independent, anatomic quantification of valve calcification
Key diagnostic criteria [13,37]	True severe AS: AVA ≤ 1.0 cm^2^ and/or mean gradient ≥ 40 mmHg with ≥20% ↑ stroke volume	Severe AS: Agatston score ≥2000 AU (men), ≥1200 AU (women)
Additional information [24,36]	Evaluates contractile reserve (predicts outcomes)	Quantifies calcification burden, predicts rapid progression and adverse events
Best use [5,7]	First-line test in LFLG AS to differentiate true vs. pseudo-severe	When DSE is inconclusive, not feasible, or contractile reserve absent
Limitations [28,40]	Requires good acoustic windows and sinus rhythm; unreliable if no contractile reserve	No functional data; radiation exposure and possible contrast use
Guideline status [5,6]	Recommended as initial evaluation in LFLG AS (Class I)	Recommended when DSE is inconclusive or discordant (Class I)
Outcome prediction [24,36]	Contractile reserve→better surgical/TAVR outcomes	High calcium score→worse prognosis and faster progression

Abbreviations: AS = aortic stenosis, LFLG = low-flow, low-gradient, DSE = dobutamine stress echocardiography, CT-CAC = computed tomography calcium scoring, AVA = aortic valve area, AU = Agatston units, TAVR = transcatheter aortic valve replacement. ↑ increase.

**Table 7:** Advanced parameters derived from stress echocardiography in aortic stenosis.

Parameter	Preferred SE Modality	Key Measurement/Definition	Clinical Use and Prognostic Value
Left ventricular systolic reserve (CR) [13,24,25,29]	DSE (low dose dobutamine)	≥20% increase in stroke volume during stress	Distinguishes true vs. pseudo-severe LFLG AS; preserved CR historically associated with lower surgical mortality; absence no longer absolute contraindication to AVR/TAVR but still signals higher operative risk
Left ventricular diastolic reserve [17,20]	ESE (exercise)	Rise in E/e′ ratio or other surrogates of filling pressure under exercise	Detects occult diastolic dysfunction; correlates with exertional dyspnea and earlier symptom development in severe AS
Transaortic flow and gradient dynamics [35]	ESE and DSE	Flow rate = stroke volume ÷ LV ejection time; gradient response to stress	Improves accuracy in inconclusive or LFLG AS; dynamic gradients reflect functional severity under physiological load
Myocardial deformation (GLS) [40,43,44,45,46,53]	ESE or DSE (with speckle tracking)	Failure of GLS to augment during stress indicates limited myocardial reserve	Early marker of LV dysfunction even with preserved LVEF; impaired or non-augmenting GLS predicts symptom onset, remodeling, adverse outcomes; prognostic in surgical and TAVR populations
Valvulo-arterial impedance (Zva) [9,44,47,48,49,50,54]	ESE or DSE	Zva = (SAP + mean gradient) ÷ stroke volume index	Integrates valvular + vascular afterload; stress-induced rise indicates poor arterial compliance, accelerated symptom onset, and worse survival; adds risk stratification beyond valve area/gradient
Volume–flow (V–Q) discordance [52]	ESE or DSE (flow-based analysis)	Mismatch between stroke volume index (SVi) and transaortic flow rate (TFR) (e.g., SVi < 35 mL/m^2^ with TFR > 210 mL/s)	Novel marker of adverse outcome; low V–Q discordance linked to better survival after TAVR; offers superior prognostic discrimination vs. SVi or TFR alone; may refine low-flow AS risk stratification

Abbreviations: AS = aortic stenosis; AVR = aortic valve replacement; CR = contractile reserve; DSE = dobutamine stress echocardiography; ESE = exercise stress echocardiography; GLS = global longitudinal strain; LFLG = low-flow, low-gradient; LVEF = left ventricular ejection fraction; LV = left ventricle; SAP = systolic arterial pressure; SVi = stroke volume index; TAVR = transcatheter aortic valve replacement; TFR = transaortic flow rate; Zva = valvulo-arterial impedance.

**Table 8:** Evolution of stress echocardiography in aortic stenosis across guidelines and consensus documents.

Document/Guideline	Year	Main Contributions	Specific Role of SE in AS	Class of Recommendation/LOE
EACVI/ASE Expert Consensus [19]	2016	First unified framework for SE beyond ischemic heart disease	Recommended ESE to unmask symptoms in asymptomatic severe AS; DSE for LFLG AS with reduced EF	Consensus document (no formal class/LOE)
Stress Echo 2020 (ABCDE protocol) [20]	2020	Introduced multiparametric protocol (A–E: wall motion, B-lines, contractile reserve, diastolic reserve, arrhythmias)	Extended SE into a holistic hemodynamic tool; highlighted potential role in valvular disease	Protocol paper (no formal class/LOE)
ACC/AHA Valve Guidelines [6]	2020	American guideline update for valvular heart disease	ESE: reasonable in asymptomatic severe AS with uncertain symptoms DSE: recommended to distinguish true vs. pseudo-severe AS in LFLG with reduced EF	ESE: Class IIa, LOE B-NR DSE: Class I, LOE B-NR
ESC/EACTS Valve Guidelines [5]	2021	European guideline update for valvular disease	ESE: for asymptomatic severe AS with equivocal symptoms DSE: for classical LFLG AS with reduced EF	ESE: Class I, LOE C DSE: Class I, LOE C
EACVI Position Papers (Imaging Toolbox) [56,57]	2022	Consolidated SE as part of multimodality imaging strategy	Emphasized multiparametric SE in discordant AS and complex cases; integration with CT-CAC and strain	Position paper (no formal class/LOE)
ABCDEG refinement for AS [20,42]	2023	Extension of Stress Echo 2020	Added systematic evaluation of transvalvular gradients (G) during stress as a dedicated AS parameter	Consensus refinement (no formal class/LOE)
ESC/EACTS Guidelines for VHD [55]	2025	Major update of 2021 guidelines; refined integrative imaging algorithm for AS and intervention thresholds	SE has an explicit role in the algorithm: DSE LFLG AS to assess flow reserve and distinguish true from pseudo-severe disease—with integration of CT-CAC (thresholds for severe AS: ≥2000 AU in men and ≥1200 in women); ESE to confirm the asymptomatic status and to identify risk markers (e.g., a sustained fall in SBP > 20 mmHg)	Evidence refers to the post-test decision, not to the test itself: Intervention may be considered in asymptomatic patients with severe high-gradient AS and LVEF ≥ 50%, if exercise testing is normal and procedural risk is low—Class IIa, Level As Intervention is recommended in symptomatic LFLG AS with LVEF < 50% after confirmation of severity—Class I, Level B

Abbreviations: SE = stress echocardiography; ESE = exercise stress echocardiography; DSE = dobutamine stress echocardiography; AS = aortic stenosis; EACVI = European Association of Cardiovascular Imaging; ASE = American Society of Echocardiography; ABCDE protocol = A: wall motion; B: B-lines; C: contractile reserve; D: diastolic reserve; E: arrhythmias; ABCDEG protocol = ABCDE protocol + G: transvalvular gradient; ACC = American College of Cardiology; AHA = American Heart Association; ESC = European Society of Cardiology; EACTS = European Association for Cardio-Thoracic Surgery; LFLG = low-flow, low-gradient; EF = ejection fraction; CT-CAC = computed tomography calcium scoring; AU = Agatston units; SBP = systolic blood pressure; LOE = level of evidence.

## Data Availability

Data sharing is not applicable to this article as no datasets were generated or analyzed during the current outline of advances in our field of expertise.
